# Supplementary material for: Integration of a Physiologically Based Pharmacokinetic and Pharmacodynamic Model for Tegoprazan and Its Metabolite: Application for Predicting Food Effect and Intragastric pH Alterations
Source: Pharmaceutics. 2022 Jun 18;14(6):1298. doi: 10.3390/pharmaceutics14061298 (PMC9230797; doi:10.3390/pharmaceutics14061298)
Supplement: Supplementary file 1 [file pharmaceutics-14-01298-s001.zip › pharmaceutics-1745342-supplementary.pdf]

pharmaceutics

Supplementary Materials: Integration of a Physiologically Based Pharmacokinetic and Pharmacodynamic Model for Tegoprazan and Its Metabolite: Application for Predicting Food Effect and Intragastric pH Alterations

Hyeon-Cheol Jeong, Min-Gul Kim, Zhuodu Wei, Kyeong-Ryoon Lee, Jaehyeok Lee, Im-Sook Song and Kwang-Hee Shin

Table S1. Intrinsic clearances using recombinant CYPs for tegoprazan and M1

	Slope	Half-life (min^{-1})	Intrinsic clearance (μL/min/pmol)
Tegoprazan			
CYP3A4	-0.031	22.68	0.86
CYP2C19	-0.011	63.20	0.61
CYP2C8	0.002	330.1	0.06
CYP2C9	-0.005	135.9	0.14
CYP2D6	<0.001	1,733	0.02
CYP2E1	-0.001	693.2	0.03
M1			
CYP3A4	-0.018	37.68	0.51
CYP2C19	0.002	303.9	0.13
CYP2C8	-0.001	577.6	0.03
CYP2C9	-0.004	182.4	0.11
CYP2D6	0.003	210.0	0.19
CYP2E1	0.001	866.4	0.02

Table S2. The predicted arithmetic mean metabolized fraction, absorbed fraction and surface solubility for tegoprazan after single administration of 50 mg tegoprazan in fasted state or at 30 min after high-fat meal.

	**Fast (n=100)**	**At 30 min after high-fat meal (n=100)**
Absorbed fraction		
Duodenum	0.33 ± 0.05	0.57 ± 0.09
Jejunum I	0.44 ± 0.05	0.35 ± 0.06
Jejunum II	0.17 ± 0.03	0.09 ± 0.05
Ileum I	0.06 ± 0.01	0.02 ± 0.02
Ileum II	0.02 ± 0.01	0.01 ± 0.01
Ileum III	0.01 ± 0	< 0.01
Ileum IV	< 0.01	< 0.01
Colon	< 0.01	< 0.01
Metabolized fraction		
Duodenum	0.01 ± 0.01	0.01 ± 0.01
Jejunum I	0.01 ± 0.01	< 0.01
Jejunum II	< 0.01	< 0.01
Ileum I	< 0.01	< 0.01
Ileum II	< 0.01	< 0.01
Ileum III	< 0.01	< 0.01
Ileum IV	< 0.01	< 0.01
Colon	< 0.01	< 0.01
Surface solubility at steady-state		
Stomach	964.1 ± 104.3	1,107 ± 129.2
Duodenum	631.6 ± 164.4	886.0 ± 198.0
Jejunum I	456.4 ± 150.7	443.6 ± 123.0
Jejunum II	403.6 ± 102.1	328.5 ± 72.61
Ileum I	261.1 ± 81.69	267.0 ± 65.68
Ileum II	204.2 ± 79.05	212.7 ± 63.22
Ileum III	175.8 ± 75.98	170.7 ± 55.10
Ileum IV	168.0 ± 74.51	157.9 ± 55.40
Colon	118.8 ± 115.2	124.1 ± 115.8

All the parameters were presented as arithmetic mean ± standard deviation.

Table S3. The predicted gastric lag time and mean residence time for tegoprazan after single administration of 50 mg tegoprazan in fast state or at 30 min after high-fat meal.

	**Fast (n=100)**	**At 30 min after high-fat meal (n=100)**
Fluid and Dissolved Drug		
Gastric lag time (h)	0	0
Gastric MRT (h)	0.27 ± 0.09	0.73 ± 0.62
Small intestine MRT (h)	3.39 ± 0.30	4.07 ± 1.38
Colon MRT (h)	45.16 ± 24.02	43.56 ± 21.74
Fine Particles		
Gastric lag time (h)	0	0
Gastric MRT (h)	0.27 ± 0.10	0.72 ± 0.60
Small intestine MRT (h)	3.40 ± 0.31	3.91 ± 1.07
Colon MRT (h)	44.23 ± 22.38	45.34 ± 22.87

MRT: mean residence time

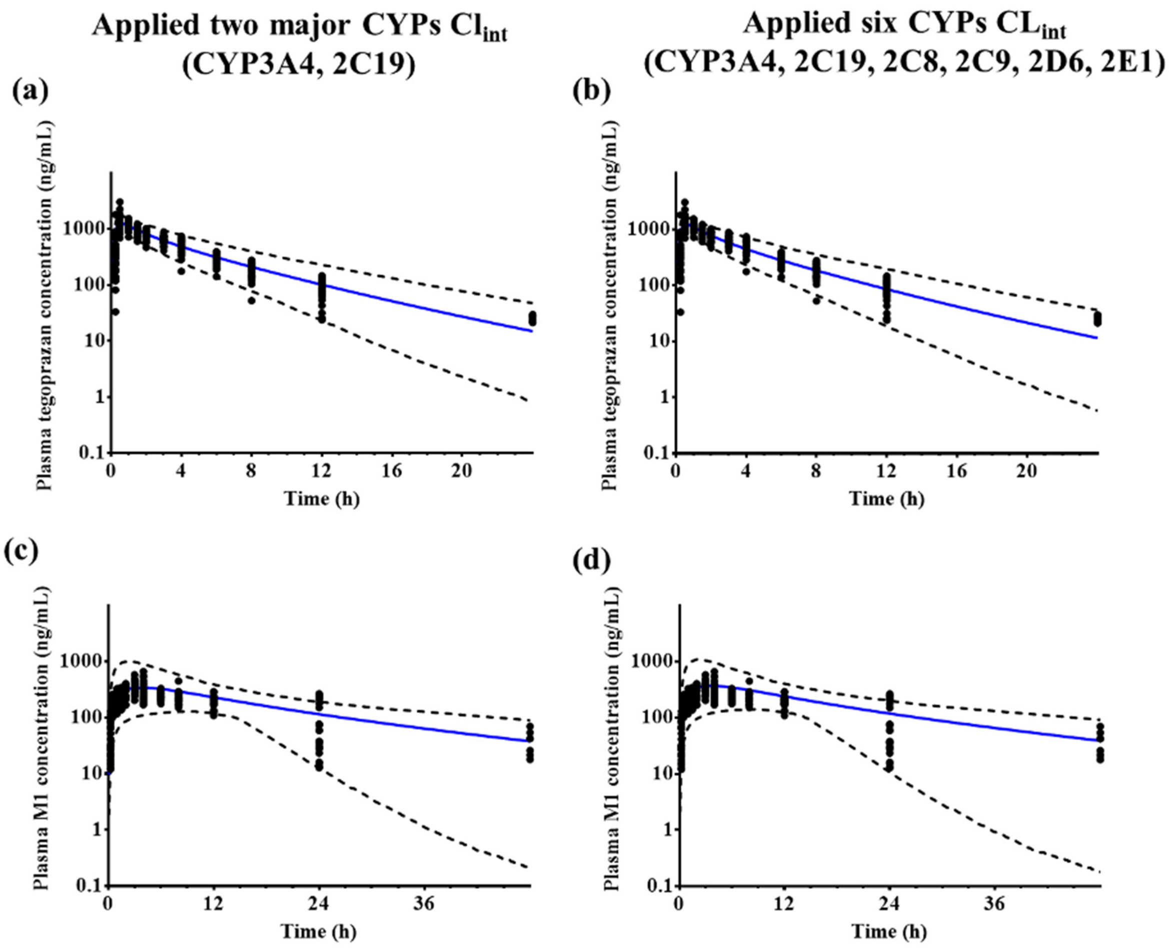

Figure S1. Predicted plasma (a, b) tegoprazan and (c, d) M1 concentration-time profiles after a single administration of 100 mg of tegoprazan. Blue solid lines and black dotted lines represent predicted mean concentrations and their 95th and 5th percentiles, respectively. Two major CYPs represent CYP3A4 and 2C19, and six CYPs represent CYP3A4, 2C19, 2C8, 2C9, 2D6, and 2E1
